# Retraction: Enhancement of Lipid Productivity in Oleaginous *Colletotrichum* Fungus through Genetic Transformation Using the Yeast *CtDGAT2b* Gene under Model-Optimized Growth Condition

**DOI:** 10.1371/journal.pone.0187171

**Published:** 2017-10-23

**Authors:** The PLOS ONE Editors

The authors and editors retract this publication [1] following an investigation into concerns around the images presented in several figures. After the publication of this article, readers raised concerns about Figures 1E, 2E-F, and 3A. Specifically,

- In Figure 1E, there are similarities between the first and third lane within the panel.
- There are similarities in bands between the gels displayed in Figures 2E and 2F.
- In Figure 3A, there are areas of duplication in images within the third panel.

The concerns were brought to the attention of the authors, who provided replicate images of the same experimental sets but were not able to provide the images underlying the figures included in the article.

An institutional investigation conducted by The Indian Institute of Technology at Kharagpur confirmed the duplication of images and recommended the retraction of the article.

The preparation of the figures falls below the standard of publication; in light of these concerns and in line with the institutional recommendation, the authors and the editors retract this publication.

The authors sincerely apologize to the scientific community for the errors in the published article.
